# Linkages between the concept of nature-based solutions and the notion of landscape

**DOI:** 10.1007/s13280-023-01935-z

**Published:** 2023-11-02

**Authors:** Barbara Sowińska-Świerkosz, Joan García, Laura Wendling

**Affiliations:** 1 Department of Hydrobiology and Ecosystems Protections, University of Life Sciences in Lublin, Dobrzańskiego 37, 20-262 Lublin, Poland; 2 GEMMA-Group of Environmental Engineering and Microbiology, Department of Civil and Environmental Engineering, Universitat Politècnica de Catalunya-BarcelonaTech, c/Jordi Girona 1-3, Building D1, 08034 Barcelona, Spain; 3 Nature-Based Solutions, VTT Technical Research Centre of Finland LTD, Kemistintie 3, P.O. Box 1000, 02044 Espoo, Finland

**Keywords:** Landscape approach, Landscape-based solutions, Landscape quality, NBS up-scaling

## Abstract

As the effects of Nature-based solutions (NBS) application are usually much broader than only the area under the project implementation, it is necessary to capture the impact on these actions of landscape as well as the influence of landscape type on the NBS effectiveness. The main aim of this study was to detect linkages between the operational of NBS and the landscape dimention, based on a systematic literature review. The results showed the existence of seven linkages: (1, 2) ‘input’ and ‘output’ resulting from the consideration of landscape as a scale of NBS implementation; (3, 4) ‘stimulator’ and ‘inspiration’ based on the contribution of landscape-based management to the implementation of NBS; (5) ‘co-beneficiary’ since the implementation of NBS affects aesthetic dimensions of landscape; (6) ‘tool’ as landscape-based indicators are used to assess the impacts of NBS; and (7) ‘foundation’ as health-supporting landscapes may be considered as a type of NBS action.

## Introduction

Nature-based solution (NBS) concept has been recently defined by the Resolution on Nature-based Solutions for Supporting Sustainable Development as "actions to protect, conserve, restore, sustainably use and manage natural or modified terrestrial, freshwater, coastal and marine ecosystems, which address social, economic and environmental challenges effectively and adaptively, while simultaneously providing human well-being, ecosystem services and resilience and biodiversity benefits" (UNEA-5 2022). As derives from the definition, crucial for considering any action as NBS actions is the use of nature, which should be treated as a priority, not as an extra addition to conventional gray infrastructure (Sowińska-Świerkosz and García 2021; Wendling et al. 2021). In addition, NBS are directed to face societal challenges or resolve urgent, and usually global environmental problems (Dumitru and Wendling 2021). The provision of multiple benefits is another core idea under the NBS concept (Science for Environment Policy 2021), together with the equitable balance of trade-offs between achievement of their primary goal(s) and the continued provision of multiple benefits (IUCN 2020). Nature-based solutions are widely viewed as a means of achieving the objectives of existing and proposed European policies, including the EU Strategy on Adaptation to Climate Change (EC 2021), Urban Agenda for the EU (EC 2021), Water (EC 2000a) and Floods Directives (EC 2007), Biodiversity Strategy for 2030 (EC 2000b), and the Nature Restoration Law (EC 2022).

Given that NBS cannot be effectively managed in isolation, as ecosystems are affected by processes occurring in surrounding areas (IUCN 2020), consideration of issues, NBS interventions and their impacts at the dimension of the landscape is most appropriate for the successful application and operation of NBS. The idea of considering landscape as a scale of NBS actions derives from the fact that "rather than as an object in itself, the landscape is considered as a comprehensive principle, to which all spatial processes are inherently related" (van Rooij et al. 2021). Therefore, all NBS are always applied at some landscape (or seascape) scale (IUCN 2016). The notion of landscape in this context should be understood with respect to the European Landscape Convention (COE 2000) as "an area, as perceived by people, whose character is the result of the action and interaction of natural and/or human factors". Thus, landscape should be defined as a zone or area perceived by indigenous people as well as visitors as a whole by taken together natural and cultural components (IUCN 2020). In other words, landscape is seen as a unique synthesis between the natural and cultural characteristics of a region, and this synthesis is an important component of the European natural and cultural heritage, worthy of protection and conscious shaping. Although the notion of landscape is rooted in geography, the development of remote-sensing technologies has mainstreamed the analysis of landscapes from a bird’s-eye perspective, and as a result the term “landscape” is increasingly society oriented rather than theoretical and academic (Antrop 2013). As a result, landscape quality deals with not only structural-ecological and cultural-historical features, but at the same level of significance considers the visual and perceptual characteristics that are of high societal importance (Cassatella and Peano 2011; Sowińska-Świerkosz and Michalik-Śnieżek 2020).

With respect to the intrinsic link between the NBS concept and notion of landscape, NBS actions have a mandate to "bring more nature and natural features and processes into cities, landscapes, and seascapes" (EC 2015). Among the eight criteria to frame green/blue intervention as NBS actions, the IUCN global standards (2020) include a criterion stating that the "design of NbS is informed by scale". The question of scale is relevant regardless of the physical size of the NBS intervention, as even local scale issues may result from disturbance beyond the local scale. This question of the scale of both core issues to be addressed by NBS as well as the scope of realized or potential impacts of localized NBS actions is applicable to social (e.g., pastoralist community), ecological (e.g., recycling of nutrients), and economic (e.g., primary production value chains) domains. Even small-scale interventions undertaken within an urban environment usually affect inhabitants of the entire district or even city (Sowińska-Świerkosz and García 2022). Such a broad impact is usually called as being of the ‘landscape scale.’ As there is no single accepted definition of ‘landscape scale’ this term indicates actions that ‘covers a large spatial scale, usually addressing a range of ecosystem processes, conservation objectives and land uses’ (IUCN 2020). According to the ecological hierarchy approach ‘landscape scale’ in conservation studies is classified between ecosystem and biome level (Gutzwiller and Forman 2002). In spatial analysis studies ‘landscape scale’ refers to the level of all classes of land cover forms considered together in contrast to the singular land cover form (patch level) or a group of the land cover types of the same type (class level) (McGarigal et al. 2023; Nowosad and Stepinski 2019). In literature referring to the NBS concept, the mesoscale is considered to align with the landscape scale (Raymond et al. 2017; Sowińska-Swierkosza and Garcia 2021).

There are certain bilateral relations between the successful implantation and operation of NBS and the landscape dimension that need to be identified, named, and systematized. It is necessary to capture both the impact of NBS actions on landscapes as well as the influence of landscapes type and quality on the effectiveness of NBS projects. Regarding the first aspect, NBS are widely recognized as suitable measures to be adopted for the conservation of landscapes, in relation to both natural and cultural features (Dumitru and Wendling 2021). Thus, it is necessary to identify the types of actions and their spatial distribution, e.g., within the built environment, that are the most effective from the point of view of maintenance or creation of high quality landscapes (Sowińska-Świerkosz et al. 2021b). Such knowledge would support decision makers to select the optimal solution to be implemented, not only in relation to the improvement of socio-economic conditions within the area subject to the action, but also accounting for wider-scale impacts as well as the up-scaling potential of NBS from local to city scale. Regarding the influence of landscapes on NBS operational, these solutions can be implemented in all types of landscapes. These types can be distinguishing based on the different criteria such as state of the anthropogenic transformation (natural, rural, urban, and peri-urban areas), location (land, inland water, and marine areas) and the preservation and uniqueness gradation (outstanding, everyday and degraded landscapes) (EC 2005; Van Eetvelde and Antrop 2009). Landscape types are usually connected to a given level of landscape quality, e.g., poor ecosystem condition may be the main reason to undertake NBS action, for example floodplain reconnection with rivers. Similarly, high value landscapes may benefit from the implementation of NBS actions characterized by minimal or no level of human intervention, such as the establishment of protected areas or conservation zones, or control of urban expansion. The quality of landscape within which an NBS action is implemented may hinder or favor the implementation of a given type of solution.

The problems of NBS interaction and effectiveness on landscape scale is one of the major knowledge gaps in the NBS studies (NetworkNature report 2022). It presents a challenge for research and innovation into NBS to understanding how multiple often individually small NBS can combine to deliver jointly significant strategies on landscape scale (EC 2020; Nelson et al. 2020). To answer this question it is vital to define how landscape values (quality) affect the effectiveness of NBS actions and the types and magnitude of economic, social and environmental benefits and costs provided by NBS implementation. The first stage to do so is to identify, name and characterize linkages linkages between the operation of NBS and the landscape dimension, understood as the mutual spatial and temporal relationships, existed within current literature being the primary aim of the present study. Such a knowledge allows to indicate aspects of landscape—NBS interactions that are well documented in literature as well as these that still require further analysis.

## Materials and methods

**Fig. 1:**
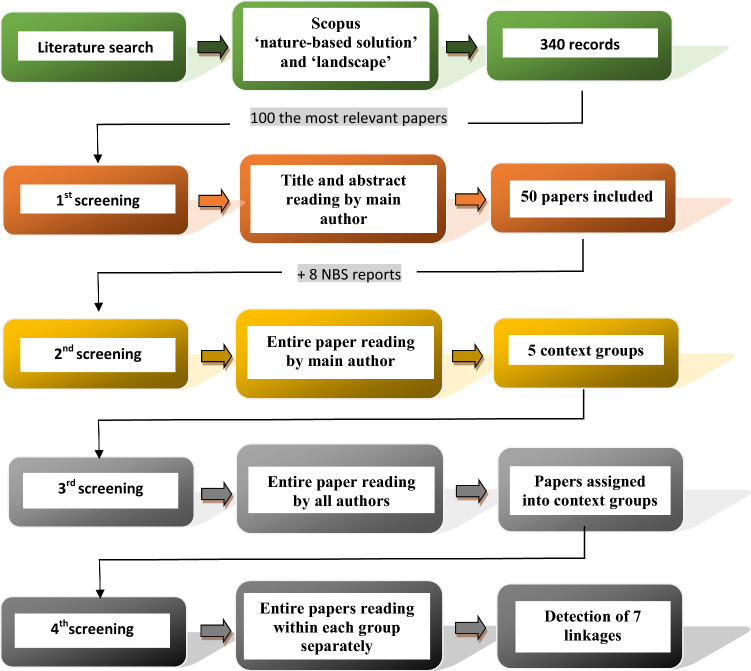
Methodological diagram of context groups and linkages detection

The method consists of five main stages (Fig. 1). First, a systematic review of literature was conducted based on the Scopus database on 20th October 2022 and the following search criteria: ‘nature-based solution’ and ‘landscape’ in title, abstract, keywords (language: English). In total, 340 records were identified, and among these 27 mentioned both search terms in the title. The entire body of the text of the 100 most relevant records were further screening (1st screening) by the main author, which showed that half of the identified manuscripts did not refer to both concepts. These 50 records either: (1) focused on NBS or landscape itself; (2) mentioned NBS as a key word without referring to it in the main body of the text; or, (3) used the term landscape only in relation to the type of area within which NBS projects were implemented (river, mountain etc. landscape). Thus, 50 publications were selected for detailed analysis. This set was expanded by eight key reports on NBS which are not included in the Scopus database but contain information concerning both of the concepts under analysis. These reports included European Commission publications (EC 2015, 2020a, b; Science for Environment Policy 2021), and reports from the International Union for Conservation of Nature (IUCN 2016, 2020), United Nations Environment Programme (UNEP 2020), and ECLIPSE (Raymond et al. 2017). In summary, 58 key documents were further analyzed. Then, these documents were secondly screening (2nd screening) by reading of the entire body of the text by the main author to detect the contexts of the use of the notion of landscape in relation to NBS actions/concept. This second screening allowed to identify five context groups, understood as the way in which term NBS was used in relation to the notion of landscape, and vice versa how the notion of landscape was used in reference to NBS. These groups were called as ‘scale,’ ‘management,’ ‘indicators,’ ‘perception,’ and ‘health.’ Then, all the authors were screening papers independently for the third time (3rd screening) and were assigning each paper to each of the detected context group. In the case of divergence among the classification, all authors decided whether assign a given paper/report to only one group or assign it to more than one group. Finally, the fourth screening (4rd screening) was executed for each group separately to detect and name linkages understood as the mutual spatial and temporal relationships between both concept under analysis.

## Results

### Contextual use of the notion of landscape in relation to NBS actions

**Fig. 2:**
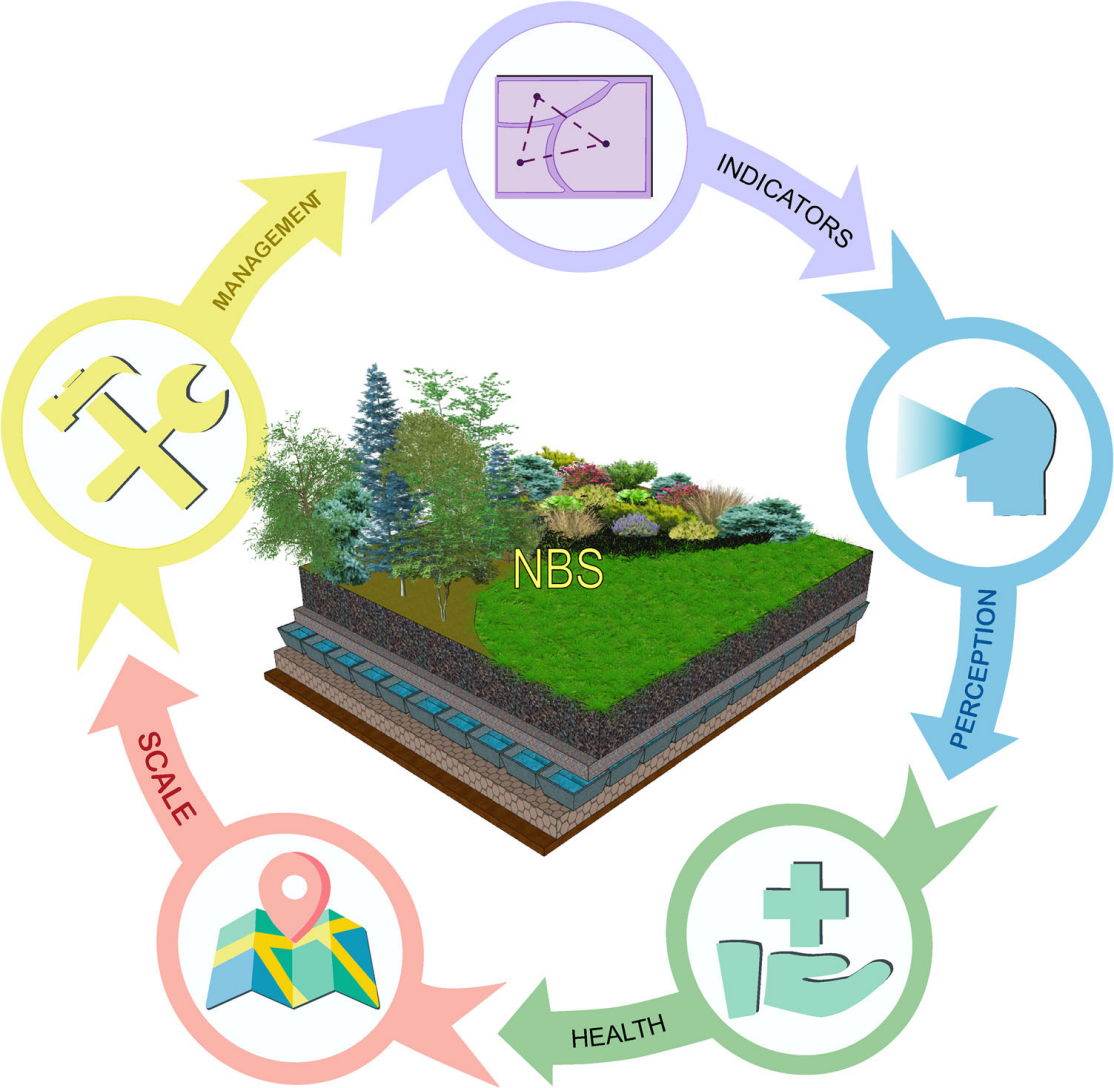
Context groups reflecting the use of the notion of landscape in relation to the NBS actions (based on 58 papers)

Results showed that the notion of landscape was used in reference to the following aspects of NBS called as a context groups (presented in the order of number of publications) (Fig. 2),

1. *SCALE: Landscape as a scale of NBS project implementation*, discussed in 19 publications (IUCN 2016, 2020; Moosavi 2017; Raymond et al. 2017; Thorslund et al. 2017; Groß et al. 2018; Guerrero et al. 2018; Quin and Destouni 2018; Carvalho Ribeiro et al. 2020; European Commission 2020a, b; Kopp and Preis 2020; Science for Environment Policy 2021; Solheim et al. 2021; van Rooij et al. 2021; Wu et al. 2021; Zandersen et al. 2021 Bunclark and Vega Hernández 2022; Sušnik et al. 2022).
2. *INDICATORS: Application of landscape-based indicators to assess the environmental impacts of NBS*, discussed in 16 publications (European Commission 2015; Fan et al. 2017; Raymond et al. 2017; Tomao et al. 2017; Thorslund et al. 2018; Makido et al. 2019; Zawadzka et al. 2019; Lee et al. 2020; Ranagalage et al. 2020; Sowińska-Świerkosz et al. 2021b; Baldwin et al. 2022; Kalantari et al. 2022; Li et al. 2022a, b; Préau et al. 2022; Schmidt et al. 2022; Vasiliev and Greenwood 2022).
3. *MANAGEMENT: Landscape-based management contributions to the implementation of NBS actions*, discussed in 14 publications (Tomao et al. 2017; Albert et al. 2019; Collier and Bourke 2020; Hundertmark et al. 2020; IUCN 2020; Plieninger et al. 2020; Frantzeskaki and Bush 2021; Gottwald et al. 2021; Mendonça et al. 2021; Puskás et al. 2021; Roggema et al. 2021; van Rooij et al. 2021; Wang et al. 2021; King et al. 2022).
4. *PERCEPTION: Landscape as an aesthetic variable of NBS perceived by people*, discussed in six publications (European Commission 2015; Calheiros et al. 2020; Gottwald et al. 2021; Li and Nassauer 2021; Wang et al. 2021; Li et al. 2022a, b).
5. *HEALTH: Health-supporting landscapes as a type of NBS action*, discussed in three publications (European Commission 2015; Dick et al. 2019; Dushkova and Ignatieva 2020).

**Fig. 3:**
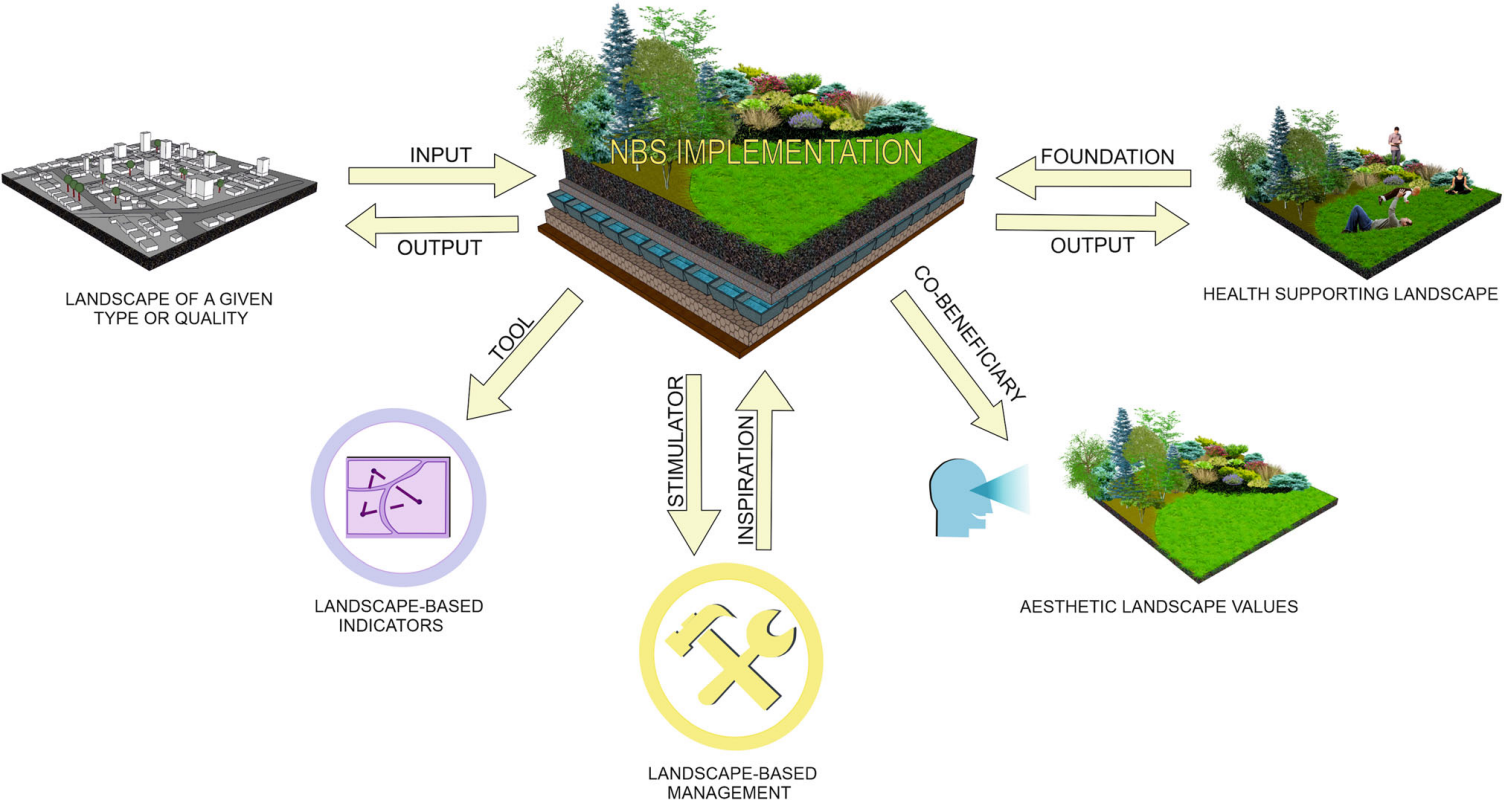
The links between context groups and the detected linkages

The fourth screening showed that, due to the homogeneity of the papers’ scope, within context groups called as ‘indicators,’ ‘health,’ and ‘perception’ only one linkage can be detected, named respectively as ‘tool,’ ‘foundation,’ and ‘co-beneficiary.’ Due to the differences in the mutual spatial and temporal relationships between both concept under analysis, within the context groups that referring to the landscape as a scale of NBS project implementation two linkages were detected: ‘input’ and ‘output’’; and within the context group that referring to the landscape-based management contributions to the implementation of NBS actions, ‘inspiration’ and ‘stimulator’ linkages were distinguished. The links between context groups and the detected linkages presents Fig. 3. The groups and linkages description is provided in “Linkages between landscape and NBS” section.

### Linkages between landscape and NBS

#### Landscape as a scale of NBS project implementation: landscape as an ‘input’ and ‘output’ of NBS actions

The present analysis showed that in the reviewed publications there are generally two perspectives of considering landscape as a scale of NBS project. From the point of view of the first perspective, the landscape-scale context resulted from consideration of NBS effectiveness as a priority. Any action ‘working with nature’ needs to be implemented at appropriate scale, which is the scale of the affected ecosystem or the ecosystem delivering certain services (Guerrero et al. 2018; van Rooij et al. 2021; Zandersen et al. 2021). Therefore, ecological restoration projects are optimally implemented at the landscape scale of in order to enhance natural ecological processes as well as to improve characteristics such as the level of structural and functional connectivity and landscape heterogeneity (Kopp and Preis 2020). According to this approach, NBS refer to actions undertaken in relation to a given spatial location, which feature the same level of landscape quality. Here, NBS actions undertaken in relation to highly urbanized landscapes or degraded seascapes can help to resolve multiple issues through the introduction of action(s) powered by nature. Such NBS actions may include, e.g., the restoration of degraded forest landscapes (Carvalho Ribeiro et al. 2020; Science for Environment Policy 2021) or the introduction of woody landscape features within dense urban areas (European Commission 2020a).

**Fig. 4:**
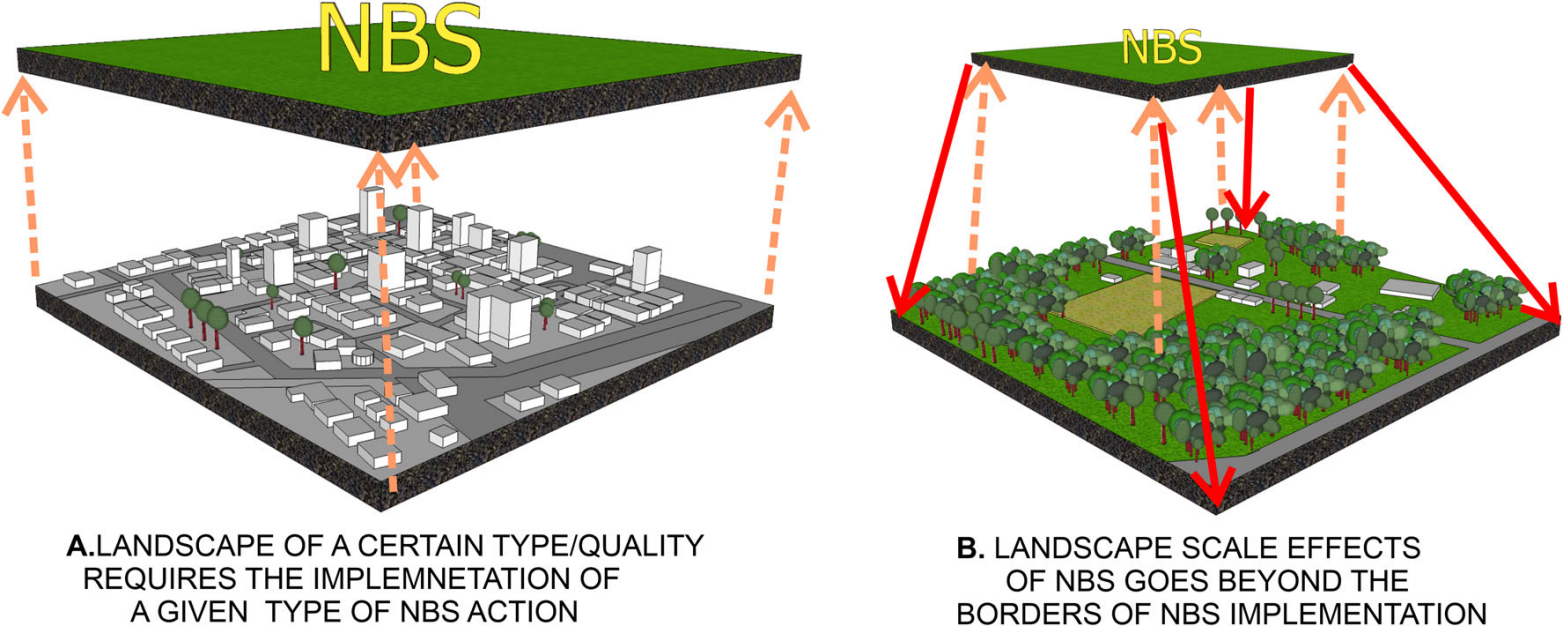
Landscape-scale effect of NBS goes beyond the borders of a solution implementation: landscape-scale as an ‘output’ of the NBS projects

From the point of view of the second perspective, both synergies and trade-offs resulting from the implementation of local/micro-scale NBS extend beyond the physical borders of the area acted upon (IUCN 2020). As a result, the impacts of solution implementation may refer simultaneously to degraded, daily life, and outstanding landscapes meaning landscapes characterized by low, moderate, and high ecological integrity or to ecotone zone(s) between them (Thorslund et al. 2017). Therefore, results of NBS should be analyzed from the point of view of the expected landscape scale effects (Groß et al. 2018) by demonstrating the effectiveness and upscaling potential of NBS interventions (IUCN 2020; Solheim et al. 2021). To do so, the effects of implementing different types of NBS across different landscape zones should be examined (Quin and Destouni 2018) (Fig. 4). These effects include ecological (e.g., biodiversity), social (e.g., knowledge and social capacity), and economic (e.g., mean land/property value) outcomes.

In summary, it may be concluded that with respect to the landscape-scale context there are two linkages between the landscape and NBS. In the first, the landscape scale is perceived as an ‘input’ to the NBS project, i.e., landscapes of a certain level of quality require the implementation of NBS actions. In the second, the landscape-scale is perceived as an ‘output’ of the NBS project, i.e., the implementation of an NBS action yields broader landscape-scale impacts.

#### Landscape-based management contributions to the implementation of NBS actions: landscape planning and governance to ‘inspire’ and ‘stimulate’ NBS actions

The analysis revealed two dominant perspectives dealing with the contribution of landscape-based management to the implementation of NBS actions. The first perspective refers to the fact that landscape planning and governance provide tools for the identification of strategies that employ NBS to address various societal challenges (Albert et al. 2019; Collier and Bourke 2020), also including the participatory approaches (Puskás et al. 2021). Because adaptive (co)management is a core characteristic of NBS, the direct application of traditional landscape management models as models of NBS management is challenging; however, are more readily managed using landscape management models. In contrast to green/blue infrastructure and similar elements of low-impact design or sustainable urban water management, for example, NBS actions are characterized by a high degree of site-specificity a well as stakeholder engagement in long-term monitoring and management. As a result, an adaptive management model must be adopted to ensure the flexible and locally adapted governance of NBS actions (IUCN 2020). Nevertheless, landscape provides both the physical and the perceived baseline for spatial development both in space and time (Nassauer 2012). Albert et al. (2019) present a consistent viewpoint, emphasizing that it is a task of landscape planning to include NBS as one type of solution. Wang et al. (2021) called landscape planning an ‘inspiration’ for NBS planning. So-called ‘landscape-based’ planning principles that mainly derives from the landscape architecture discipline offer a common ground for specialists of different disciplines and as such may be treated as a basis for the management of NBS projects (King et al. 2022). The IUCN (2020) global standards emphasize that the long-term operation and monitoring of NBS actions requires a landscape/seascape-scale approach. Van Rooij et al. (2021) further expand upon this idea through use of the term ‘landscape-based planning’ rather than ‘nature-based solutions.’ Behind such an approach lies the fact that according to these authors, “landscape” is relatively more multi-dimensional than “NBS,” intrinsically embodying biophysical, social, and cultural elements and thus ensuring the evaluation of the full range of factors affecting the performance of the implemented solution. Therefore, many landscape strategies can be interpreted as NBS, including restoration and conservation actions (Albert et al. 2019) and agroforestry actions (Plieninger et al. 2020).

The second perspective follows the fact that NBS actions do not only draw from the landscape-based planning and governance, but their effectiveness may be favored or hindered due to different landscape management choices (Hundertmark et al. 2020; Mendonça et al. 2021). As such, a long-term future landscape vision may be considered as a reason to undertake NBS projects (Gottwald et al. 2021), or as stated by van Rooij et al. (2021) as a *pathway towards a defined future*. This is well justified, as revealed by Wang et al. (2021), because NBS paradigms are consistent with the expectations of different actions and visions for the future of physical landscapes. Therefore, the implementation of sustainable landscape management, which promotes multiple benefits, including multi-stakeholder interests and multiple objectives (Plieninger et al. 2020; Frantzeskaki and Bush 2021), is an ideal means to support the effective implementation and longer-term management of NBS actions.

In summary, the linkages between the landscape and NBS may be viewed as ‘inspiration’ (after Wang et al. (2021)), i.e., the need to implement and manage NBS actions makes use of landscape-based planning and governance tools, or ‘stimulation,’ i.e., the adaptation of a given scheme of landscape-based management favors or hinders the implementation and effectiveness of a given NBS project.

#### The application of landscape-based indicators to assess the environmental impacts of NBS: landscape-based indicators ‘tool’ for the evaluation of NBS actions

The application of landscape-based indicators as a tool for analyzing environmental impacts of NBS is facilitated by the fact that NBS actions affect the structural and ecological dimensions of landscape quality by improving the connectivity of patches of greenery, promoting biodiversity, increasing ecological stability, and enhancing the natural capital (Li et al. 2022a, b; Sowińska-Świerkosz et al. 2021a, b). These aspects may be measured based on the application of so-called landscape-based surrogates, i.e., indicators based on the structure and configuration of patches of land cover forms as well as spectral variability between bands which indirectly attest to the ecological state of a given area (Sowińska-Świerkosz and Michalik-Śnieżek 2020). Surrogate measures provide only approximate information on the ecological quality of a given area but are of high importance when other data types are unavailable or available only at high cost and/or significant time or effort. Such a situation is typical for many types of NBS due to their innovative character and/or spatial extent (Sowińska-Świerkosz and García 2021). Among the landscape-based surrogates used to assess the effectiveness of a given NBS action, the following may be highlighted: (1) land use and land distance measures (Wang et al. 2021); (2) spatio-temporal changes (European Commission 2015; Fan et al. 2017); (3) microclimate modeling (Makido et al. 2019; Ranagalage et al. 2020); and, (4) indices showing the ratio of the area covered by green and blue space (Raymond et al. 2017; Tomao et al. 2017). Besides, landscape indices are used to identify optimal NBS localization and areas (Baldwin et al. 2022; Kalantari et al. 2022; Préau et al. 2022; Schmidt et al. 2022).

Another important aspect of the application of landscape-based indicators results from their ability to provide information on the type and quantity of ecosystem services provided by NBS actions. Among these services, coastal protection, biological diversity, groundwater storage and soil moisture regulation, flood regulation, and contaminant retention encompass many of the core benefits desired as an outcome of NBS actions (Thorslund et al. 2018). The evaluation of these and similar landscape-scale indicators is also critical for the assessment of synergies and co-benefits provided by NBS (Zawadzka et al. 2019).

Surrogates based on landscape data should, however, constitute only one type of indicator used to assess the overall performance or impact of an NBS action (Sowińska-Świerkosz and García 2021). Social and economic information obtained via, e.g., workshops, surveys, and epidemiological/statistical data sources are needed to estimate the socio-economic resilience of NBS actions (Lee et al. 2020). In conjunction, various types of environmental indicators including the reduction of greenhouse gas emissions, and carbon removed or stored in vegetation and soil, among others, can support assessment of the effectiveness of NBS actions with respect to defined targets (Dumitru and Wendling 2021). Governance and economic indicators are useful to assess the practical aspects of NBS implementation, also impact and longer-term sustainability. The selection and application of a suite of appropriate indicators across social, environmental, and economic domains supports the clear identification of issues and targets, and informs the selection of the optimal NBS action to be implemented while taking into account local conditions. Thus, a range of performance and impact indicators based upon landscape data, in situ measures and baseline assessment are required for the evaluation of the overall effectiveness of any NBS action.

In summary, this linkage between the landscape and NBS can be named ‘tool,’ i.e., the need to employ landscape-based indicators in the evaluation of the performance and impact of NBS actions.

#### Landscape as an esthetic variable of NBS: visual landscape dimension as ‘co-benefit’ of NBS actions

To understand the NBS impacts on visual landscape dimensions, the statement made by Wang et al. (2021) is crucial: “nature’s contribution to people is literally understandable and communicable to lay people in conveying and imaging the connection between nature and the good quality of life.” This means that people expect landscapes with high visual appeal rather than landscapes of high ecological values. As a result, when people engage in NBS actions that transform landscapes, adjusting them to their needs, at the same time the appearance, esthetic appeal, and compositional values of landscapes affect the well-being, attitudes, and needs of people (Plieninger et al. 2013). Considering that one of the core concepts of NBS is the inclusion of stakeholders views and needs (EC 2015; Gottwald et al. 2021), any solution considered an NBS should positively impact perceived value (Calheiros et al. 2020), and thus increase the attractiveness of landscapes and cities (European Commission 2015).

Tangible elements of NBS may be both positively and negatively perceived by people. In general, green and blue natural elements positively affect the visual values of landscapes (Wang et al. 2021), whereas elements of gray infrastructures, such as pipes, wind turbines, and artificial surfaces are typically viewed negatively. Esthetic effects of NBS of innovative character, such as energy self-sufficient buildings equipped with green roofs, solar panels, and rainwater recycling equipment, however, are not well known (Sowińska-Świerkosz and Soszyński 2019). Such innovative solutions may be used to replace outdated or neglected elements of technical infrastructure, thus improving the visual dimensions of the landscape. However, technical solutions with blue-green features (e.g., integrated blue-green-gray systems) include elements which differ from well-known forms and shapes and thus, although “green,” may be perceived as being inconsistent with local traditions or values, and not fitting the desired image of a given place. For example, it was found that generally smart NBS may degrade everyday experiences of urban nature when they introduce noticeable landscape change (Li et al. 2022a, b; Li and Nassauer 2021).

In summary, this linkage between NBS and landscape can be called ‘co-beneficiary’: the implementation of NBS actions enhance the landscape esthetic value and positively influence its perception by observers.

#### NBS positive impact on human well-being: NBS actions as a ‘foundation’ of health-supporting landscape

Although, health-supporting landscape main aim is not the therapy (in contrast to the healing landscape and therapeutic gardens what aim to provide relief from physical symptoms, illness or trauma (Williams 2017)) a growing body of empirical evidence shows that green and blue spaces, including NBS affect well-being and health by mediating exposure to potentially harmful factors, enabling psychophysiological stress recovery and attention restoration, fostering social connectivity and contributing to reduced chronic disease (Sullivan and Chang 2017; Kabisch and Haase 2018).

**Fig. 5:**
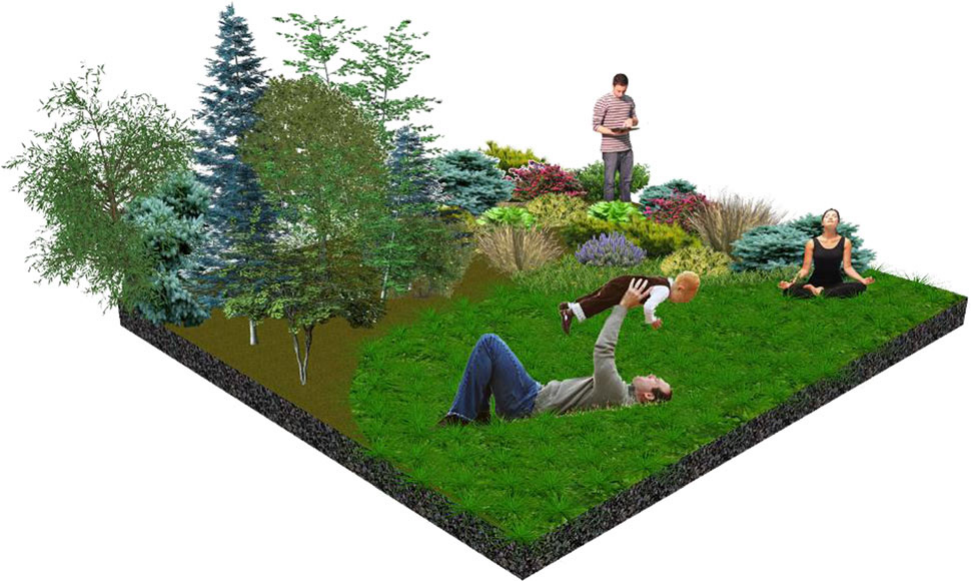
Health-supporting landscapes as a type of unsophisticated NBS actions of positive impact on mental and physical health

There are many types of NBS which have the potential of being considering as the foundation of health-supporting landscape (EC 2015). Among them, Dushkova and Ignatieva (2020) listed: public gardens, community gardens, sport- and playgrounds, allotment gardens, healing gardens, walking in special routes, and touching/smelling gardens. These solutions certainly possess one of the key features decided on the consideration of any solution as an NBS: there provide multiple benefits simultaneously, including supporting health and well-being (Dick et al. 2019). Among them can be listed: the possibility of spending time outdoor, re-connecting people with nature, the motivation to start physical exercises, the reduction of depression, and the reduction of the number of heat-related deaths (European Commission 2015; Sowińska-Świerkosz et al. 2021a) (Fig. 5).

The consideration of the existing elements of green and blue infrastructure (GBI) as NBS of health-supporting landscape scale, however, may be questioned in relation to several aspects. First of all, they usually lack the intrinsic stakeholder engagement in defining these solutions. Secondly, the aspects of biodiversity gain are in most cases not taken into account and there is a real lack of information about biodiversity, degree of “wildness”/naturalness, etc. and how these important factors influence restorative capacity of public green spaces. Thirdly, these elements of infrastructure requiring the high costs of management (the cost of a solution’s implementation, management, monitoring, and damage over a certain timeframe should not exceed the potential environmental and social benefits), and fourthly they are being of law ecological effectiveness (NBS should promote the renewable sources of energy, the use of rainwater or treated water instead of drinking water to irrigate and maintain solutions, and the re-use of materials) (Sowińska-Świerkosz et al. 2021a). As a result, although health-supporting landscape interventions could be the outcome of an NBS action, only those that have followed a systematic process consistent with the IUCN (2020) principles and meet the requirements of NBS per the UNEA-5 (2022) definition can be labeled an NBS.

In searching for the linkage between the notions of landscape and NBS for human health and well-being promotion, it may be concluded that the concept of NBS have the potential to become the theoretical foundation for health-supporting landscape NBS.

## Discussion

### The landscape characteristics impact on the selection and operational of NBS actions

As shown by the results of the present analysis, landscape affects the selection and operation of NBS actions as a visual and functional sum of environmental and cultural context. Given landscape characteristics generally necessitate the implementation of a particular type of NBS solution (Sowińska-Świerkosz and García 2021); however, natural characteristics of the landscape should be treated as opportunities rather than limitations for the implementation of NBS (van Rooij et al. 2021). For example, minimal intervention with a view to better use and sustainably manage ecosystems is recommended in those systems featuring high or unique ecological and cultural values. In highly modified systems or ecosystems with compromised integrity, more intensive intervention may be required to restore ecological integrity and the delivery of ecosystem services. For example, urban ecosystems can typically be regarded as highly modified from their natural state and relatively more intensive interventions, such as the actions undertaken for long-term management of urban green spaces, are generally required to develop sustainable and multifunctional ecosystems and landscapes that sustainably deliver multiple benefits (Dumitru and Wendling 2021). Degraded landscapes in particular should be addressed via multifacted actions, which include ecological, social, and economic regeneration. This multi-pronged approach, which builds upon the core pillars of NBS actions, supports the successful achievement of sustainable place regeneration by developing or enhancing people-nature connections, minimizing use of environmental resources, enhancing place resilience to natural disasters, and strengthening social cohesion (Xiang et al. 2017).

Some actions which are particularly focused on peri-urban areas (e.g., controlling urban expansion), agricultural landscapes (e.g., polycultures and agroforestry systems), or coastal areas (e.g., protection or restoration of mangroves or seagrass beds), clearly necessitate landscape-scale management approaches. Site evaluations, implemented actions, and management plans must be tailored to each unique social-ecological context, including understanding of how stakeholders value and interact with local landscapes as well as the full range of challenges faced. Locally implemented NBS can respond to urgent global challenges, e.g., through actions targeting climate change adaptation and mitigation, improved management of social, ecological, and economic vulnerabilities and risks, or long-term water security (EC 2015). Landscape type and quality has an impact on the effectiveness of implemented NBS actions. The consideration of local environmental conditions, such as climate, soil type, local biodiversity, and humidity, is critical for successful implementation and operational phases (Xing et al. 2017), along with consideration of how these environmental factors may change with a changing global climate. It is especially important to consider in NBS actions the landscape-scale functional and structural connectivity of ecosystems as well as potential changes in the distribution of plant and animal species.

When accounting for the impact of landscape type on the selection and performance evaluation of NBS actions, a ‘case-by-case’ approach should be adopted, rather than a ‘copy-paste’ approach (Sowińska-Świerkosz and García 2021). NBS effectiveness is very context specific, depending on the societal challenges being addressed, ecosystem types, specific landscape/seascape characteristics, the socio-economic-cultural system and the composition of stakeholder groups (IUCN 2020). The specific sum of these features may hinder of favor the implementation and successful performance of a given NBS, meaning that there are a range of potentially suitable solutions that may be implemented in relation to a landscape of a particular type or quality to achieve a given objective or outcome.

### The impact of NBS implementation and operational on landscape quality and values

Objectives under the Biodiversity Strategy to 2030 and the proposed Nature Restoral Law highlight the need for NBS actions to be designed, implemented, and evaluated in the context of the wider landscape. Through the protection, conservation, restoration, and/or sustainable use and management of ecosystems, NBS actions can be expected to deliver a multitude benefits, and to promote good-quality landscapes and seascapes by restoring or maintaining the integrity of terrestrial and aquatic ecosystems (EC 2015). These benefits inter alia include the improvement of habitat connectivity, maintenance or restoration of ecological processes and enhancement of ecosystem stability, and the increased diversity and continuity of ecological structures and processes. The NBS of the highest positive impact are ‘landscape-scale’ initiatives, such as regional/national strategies for afforestation or flood protection that affect broad spatial scales (Sowińska-Świerkosz et al. 2021b). Ecosystem restoration actions applied in highly degraded areas in need of regeneration have substantial potential for a high degree of positive impact through the improvement of ecosystem integrity from low to moderate or high.

The most commonly mentioned dimension of impact of NBS on landscape quality among the publications analyzed dealt with the esthetic, rather than ecological, dimension. Visual landscape values are often viewed as the added-value of the implementation of NBS actions. This aspect is of critical importance as NBS actions must be accepted by diverse groups of users, and visual/esthetic value is a key factor of public assessment (Wang et al. 2021). Therefore, in NBS project directed to urban regeneration strong emphasis is given to perceived values of landscape, e.g., through the use of flowering and multi-color plants. The perceived value or impacts of NBS may have a multisensory character. For example, greenery may be used as a natural acoustic screen, wetlands and forest areas attract singing birds, flowering trees and blooming flowers provide a pleasant fragrance, and fruit-bearing trees and plants can yield tasty snacks.

It is important to understand the synergies and trades-off at the landscape scale that may be generated by NBS actions (IUCN 2020). The recognition of such linkages among the benefits provided, however, is one of the more difficult aspects of NBS assessment. First, it is not possible to comprehensively evaluate all the potential benefits and trade-offs of any given intervention a priori, due at least in part to the unique context of each NBS action (Nesshöver et al. 2017). Secondly, some synergies and trades-off may occur within a short time, wherein realization of others may require a much longer timeframe (Sowińska-Świerkosz and García 2021). At present, there is limited available information concerning the systematic mapping of synergies and trade-offs between different categories of the impacts of NBS (Dumitru et al. 2020). To consider the full set of regulating, provising, and cultural ecosystem services generated by NBS actions, and relations among them, the IUCN (2020) global standards recommended that each NBS must be developed in the context of the wider ecosystem through landscape/seascape planning. Such approach ensure that solutions are strategic and maximize benefits to both people and ecosystems, while minimizing adverse effects on adjacent ecosystems and human populations.

A given NBS project may affect both the landscape quality of the area directly acted upon, as well as the surrounding areas which have not been directly subject to the action. As a result, monitoring of NBS performance may not capture the full range of benefits and trade-offs of a given intervention on the landscape—e.g., district or cityscape, quality (Sowińska-Świerkosz et al. 2021b). Therefore, monitoring and understanding how NBS performance and impacts evolve with time and at broader, e.g., catchment or landscape, scale provides key insights into their respective potential for up-scaling (Dumitru and Wendling 2021). The scaling of NBS, however, requires new forms of planning and governance approaches to embrace cross-sectoral dialogue and collaboration, and citizen participation (Frantzeskaki et al. 2019). The direct implementation of existing landscape management tools to implement and assess innovative NBS actions may not be suitable, as essential elements of social-ecological ecosystems may be overlooked; however, existing landscape management tools and practices can offer validated ‘best practices’ to be incorporated within detailed and standardized monitoring methods, reporting protocols and guidance at the different stages of the NBS life cycle.

### Differences between the landscape-based and NBS based approaches

There are strong links between the landscape approach and nature-based solutions approach which encourages an integrated approach to land management, considering the costs and benefits of land use decisions, and pursuing those that minimize risks and maximize opportunities for people, for nature and for the economy (IUCN 2020). Despite of this fact, there is lots of evidences and published papers discussing the differences among these two approaches. Such discussion is required to distinguish NBS intervention from other interventions from the green concept family. Not each green/blue solution should be considered as NBS—only those that have followed a systematic process consistent with the IUCN (2020) principles and meet the requirements of NBS per the UNEA-5 (2022) definition can be labeled an NBS.

From the conceptual point of view, there are certain differences between landscape-based management and NBS concepts that depend on the main ‘pillars’ to which they refer. Although both landscape management and NBS concepts refer to a socio-ecological system that provides human well-being benefits (Dick et al. 2019; Sowińska-Świerkosz and Michalik-Śnieżek 2020), NBS embrace environmental, social, and economic pillars (Parker and de Baro 2019; Sowińska-Świerkosz et al. 2021a; UNEA-5 2022), whereas the landscape has spatial, ecological, historical-cultural, social, and perceptual characteristics (Medeiros et al.^2021^; Sowińska-Świerkosz and Michalik-Śnieżek 2020). As a result of concept framing, the NBS concept and definition highlight their effectiveness and efficiency orientation, while the concept of landscape has a more perceptual orientation. As a result, successful NBS projects must be *inter alia* characterized by sustainable implementation and maintenance costs. Balancing costs and benefits and adaptively managing trade-offs throughout the NBS life-cycle in order to deliver desired outcomes at reasonable cost is one factor that distinguishes NBS actions from similar green and blue solutions (Sowińska-Świerkosz and García 2022). According to the IUCN, NBS actions should exhibit costs comparable to or lower than other possible solutions to the same societal challenge(s) (IUCN 2020). In contrast, the relative success of actions implemented based upon a landscape amenity approach is largely dependent upon the harmonious combination of natural and anthropogenic elements to elicit a positive perception of a given landscape/seascape. In addition, NBS actions are differentiated from landscape-based solutions in terms of the relative emphasis of NBS actions on technical feasibility, political desirability, long-time sustainability, and scaling for optimal benefit (Science for Environment Policy 2021; Sowińska-Świerkosz and García 2021). The traditional landscape management does not intrinsically seek to optimize economic or social benefit alongside environmental benefit. Really traditional landscape management is all about the environment, and ecosystem integrity.

Regarding the practical point of view, the term ‘solution’ used in reference to NBS actions is critical as it implies that a particular challenge or problem should be solved (Albert et al. 2019): NBS promote the *idea of nature bringing a solution* (Hanson et al. 2020). The notion of landscape, on the other hand, is not connected to challenge orientated issues. Of course, a given level of landscape quality or a given model of landscape-based management may be connected to (a) particular environmental problem(s), but this is not obligatory.

A number of existing and proposed international policy initiatives targeting major societal challenges, including climate change, biodiversity loss, and sustainable development, act as drivers for the expansion of the NBS concept to more explicitly encompass concepts of landscape and best practices from landscape management approaches. The view of landscape as a complex social-ecological system is consistent with the concept of NBS as actions to simultaneously address multiple concerns and to deliver multiple benefits across social, economic, and environmental domains. In particular, the consideration of landscape units within NBS actions, e.g., conservation, restoration, and sustainable management at large spatial scale, is inherent within the NBS concept, which focuses on ecosystems and their services.

## Conclusion

The conducted study contributes to one of the major knowledge gaps in the NBS studies that referring to the NBS interaction and effectiveness on landscape scale. It allows to identified seven primary linkages between the NBS and the landscape (called as ‘input,’ ‘output,’ ‘stimulator,’ ‘inspiration,’ ‘co-beneficiary,’ ‘tool,’ and ‘foundation’) being one of the first steps to understand NBS-landscape interactions. The results showed that landscape type, its ecological quality and local environmental conditions to the greatest extend influence the selection and performance of NBS and that the implementation of NBS affects the ecological and perceptual integrity as the landscape scale.

We conducted an investigation based on the existing published body of knowledge which already had a review process. While expert interviews can be valuable, we believe that our choice was appropriate considering the objective and the scope of our research. However, we acknowledge the value of interviews and that could be a valuable tool for future research in order to complement and expand our findings.

From the point of view of the international contributions of the study, regardless of the geographical context, it was showed that the impacts of NBS implementation on ecological and visual landscape quality is well documented within the scientific literature. Further exploration, however, is needed of landscape scale synergies and trades-off generated by NBS actions of various type and scale. As a result of their complex character, both NBS actions and landscape studies need to be based on multi-factor, interdisciplinary, and intersectoral approaches. Each discipline and field of practice brings unique processes, tools, and perspectives, which must be integrated to evaluate the effectiveness of NBS in the context of diverse dimensions of landscape quality.

From the management point of view, results showed that the landscape-based management and indicators positively contribute to the implementation of NBS actions, provided the application of adaptive management approach and the stakeholder engagement in long-term monitoring and management. What have to be changes in the NBS studies is to demonstrate the effectiveness and upscaling potential of NBS interventions to include both, ecological, social and economic outcomes beyond the area under the implementation.
